# Addressing data privacy in matched studies via virtual pooling

**DOI:** 10.1186/s12874-017-0419-0

**Published:** 2017-09-07

**Authors:** P. Saha-Chaudhuri, C.R. Weinberg

**Affiliations:** 1 Department of Epidemiology, Biostatistics and Occupational Health, McGill University, 1020 Pine Avenue West, Montreal QC, Montreal, Canada; 2 Biostatistics and Computational Biology Branch, National Institutes of Environmental Health Sciences, NIH, 111 T.W. Alexander Drive, RTP, Durham, NC USA

**Keywords:** Data privacy, Matched case-control design, Conditional logistic regression, Specimen pooling, Distributed data network

## Abstract

**Background:**

Data confidentiality and shared use of research data are two desirable but sometimes conflicting goals in research with multi-center studies and distributed data. While ideal for straightforward analysis, confidentiality restrictions forbid creation of a single dataset that includes covariate information of all participants. Current approaches such as aggregate data sharing, distributed regression, meta-analysis and score-based methods can have important limitations.

**Methods:**

We propose a novel application of an existing epidemiologic tool, specimen pooling, to enable confidentiality-preserving analysis of data arising from a matched case-control, multi-center design. Instead of pooling specimens prior to assay, we apply the methodology to virtually pool (aggregate) covariates within nodes. Such virtual pooling retains most of the information used in an analysis with individual data and since individual participant data is not shared externally, within-node virtual pooling preserves data confidentiality. We show that aggregated covariate levels can be used in a conditional logistic regression model to estimate individual-level odds ratios of interest.

**Results:**

The parameter estimates from the standard conditional logistic regression are compared to the estimates based on a conditional logistic regression model with aggregated data. The parameter estimates are shown to be similar to those without pooling and to have comparable standard errors and confidence interval coverage.

**Conclusions:**

Virtual data pooling can be used to maintain confidentiality of data from multi-center study and can be particularly useful in research with large-scale distributed data.

## Background

Each year in the EU, Canada and elsewhere, regional and federal governments and health care authorities collect an immense amount of personal data on health care use, diagnoses, risk factors and behaviors. The advent of personalized medicine and the genomic revolution will result in the further gathering of large quantities of very sensitive data for large segments of the population. Although these data could provide critical information for the management of the health care system, for studying causation of diseases, prognosis and the impact of treatment or prevention efforts, their use is constrained by legitimate concerns about privacy. While data confidentiality is not entirely a new issue, of late it has attracted the attention of many epidemiologists, due to the new EU General Data Protection Regulation effective from 2018 [1]. After a fierce debate and legitimate pushbacks from the research community [1–9] the current version of the regulation only requires broad consent in “certain areas of research when in keeping with recognized ethical standards” [10] and an exemption for medical research carried out in “public interest” [7]. The prominence of distributed data networks such as FDA Sentinel and CNODES [11] has also brought the issue of data privacy into the forefront of clinical and epidemiological research.

Most standard data analysis techniques require individual data (also known as microdata) for estimation and inference; thus, they are not applicable in a setting where microdata cannot be shared without risking disclosure of sensitive information. Frequently, data for research undergo anonymization to remove identifying information. However, one may still be able to identify participants using combinations of variables (e.g. if there is a unique person of a certain age, diagnosis, body mass index, town of birth) or auxiliary information available from other sources [12, 13]. Sometimes the data are deliberately coarsened to obscure individual identities, leading to loss of precision and statistical power.

**Fig. 1:**
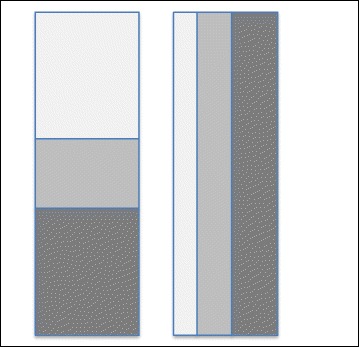
Models of horizontally and vertically partitioned data as adapted from Karr, et al. (2007). Each row is for one subject, and columns are for covariates. (**a**) Horizontally partitioned data. Data subjects are partitioned among database owners or nodes. (**b**) Vertically partitioned data. Covariates are partitioned among nodes

Privacy issues have plagued other fields as well, such as survey research, resulting in the development of analytical approaches that can extract valuable information from microdata while protecting data privacy [14–16]. Privacy-preserving statistical analyses strive to maintain a balance between data usability and confidentiality. Privacy-preserving methods have many uses, in particular for analysis of sensitive socio-economic, financial, infectious disease and genetic/genomic data. Recently, agencies generating and using “distributed data” such as disease registries or healthcare surveys where data are kept secured at the regional level and not shared between regions (see Fig. 1 for a schematic of distributed data), have been looking to adopt privacy-preserving analyses to enable analysis of the merged data while avoiding data sharing and identification of participants. Data privacy is also relevant for epidemiological research, as epidemiologic studies use secondary data analysis or reuse data for research that were collected for other purposes, e.g., routine healthcare delivery, or enter into consortial arrangements to improve power.

Current approaches to data analysis within a distributed data setting include coarsening, aggregate data sharing, distributed regression, meta-analysis based on data summary and score-based methods [17]. However, these methods have many limitations, including analytical inflexibility, large analysis burden on nodes, inability to detect subgroup associations, etc. One of the limitations of meta-analysis arises due to heterogeneity [18–20]. When the patient subpopulations differ from node to node leading to a potential interaction between the exposure of interest and nodes, meta-analysis is unsuitable to detect the interaction. A similar issue arises for detection of subgroup association under heterogeneity. Furthermore, modification of the statistical model to include or exclude covariate(s) or to modify the functional form requires each of the nodes to follow the new protocol afresh. As a result, exploratory analyses are difficult to perform with multi-node meta-analysis. Consequently, procurement of individual patient data for meta-analysis has become a strong trend in recent research [21–24]. In a meta-analysis setting, a coordinating center is usually in charge of standardizing the statistical analysis and the protocol. However, statistical analyses still pose significant burden on the nodes because analyzing the data locally as required by meta-analysis necessitates that a team capable of performing statistical analysis be present at each node. We argue that though meta-analysis is relatively straightforward to perform, without the needed statistical knowledge and know-how, it can be dangerous to run statistical analysis as a black box.

We propose a novel application of an existing epidemiologic tool, specimen pooling, for confidential analysis of data arising from distributed data networks [25]. We consider the situation where multiple agencies or nodes are each responsible for a subset of the analytical dataset and are unable or unwilling to combine the data together for statistical analysis due to privacy concerns. The goal is to devise a way to merge the data for statistical analysis that efficiently uses the information from all the agencies without sharing individual-level data.

In this manuscript, we show how specimen pooling can be adapted to pool and analyze confidential data from a matched case-control study. Matching is very common in observational studies and provides great confounder control [26]. A different distribution of confounders among subjects with a disease outcome (“cases”) versus those without the outcome (“controls”) can lead to biased association estimates between the primary exposure of interest and the outcome. In a matched design, one or more controls are matched to one (or more) case(s) to reduce the effect and the resulting bias due to the presence of confounders. When one control is matched to a single case the design is called a 1:1 matched design. More generally, when M controls are matched to N cases, the design is called *N:M* matched design. There are several other variations of matching, e.g., frequency matching, counter matching, etc. Our focus here is to employ pooling in a matched design setting as a privacy-preserving analytical strategy and demonstrate the feasibility of our approach.

We use the term “pooling” in two different contexts: “physical” pooling or mixing of biospecimens and “virtual” pooling of individual data or microdata (microaggregation), both giving rise to aggregate levels (sums) of covariate values. The idea of specimen pooling prior to assay originated during World War II [27] and has been developed extensively for infectious disease settings [28]. We focus on data pooling (rather than specimen pooling) for estimating an exposure OR using a logistic regression model for a binary disease outcome [29, 30]. Because pooling or variable aggregation masks individual data, the strategy preserves privacy. [25] We use simulations to demonstrate data pooling for analysis of matched case-control design or a matched design with a binary endpoint. The idea of specimen pooling in this context is similar to a privacy-preserving technique called microaggregation, which has been used frequently for estimating parameters of a linear regression model [25].

The rest of the manuscript is organized as follows. In the methods section, we outline specimen pooling for a matched case-control study and show how it can be adapted for data pooling. We provide simulation results and a real data example in the results section and conclude with a discussion of the strengths and limitations of the proposed approach.

## Methods

It has been shown that for a matched case-control design [26], specimen pooling and pooled conditional logistic regression model can be used to estimate parameters of an individual-level conditional logistic model [30]. For a continuous exposure *U* and a disease of interest *D* (1 for cases and 0 for controls), the contribution of the *i* ^*th*^ matched pair (1:1 matched design) is given by the following conditional logistic model:

1 $$ \frac{e^{\beta {u}_i^1}}{e^{\beta {u}_i^1}+{e}^{\beta {u}_i^0}} $$

where ${u}_i^1$ denotes the observed exposure for case and ${u}_i^0$ denotes the observed exposure for control. Here, *β* denotes the log odds ratio (OR) associated with unit increase in exposure. Additional covariates including effect modifiers (EMs) as well as multiple controls per case  can be accommodated. Standard statistical software^1^ can be used for parameter and standard error (SE) estimation.

To estimate *β* based on a matched case-control design where specimens are pooled prior to the measurement of U, a poolsize *g* is decided first [30]. Then *N/*g groups (or pools) are formed at random by partitioning the *N* matched strata into groups of *g* (assuming *N* is a multiple of *g*). Then, biospecimens from each of the *g* cases in each group are physically combined to obtain a single marker or exposure measurement using the combined specimen. Similarly, biospecimens from each of the *g* controls from the same matched strata are physically combined to obtain a single measurement for the combined specimen. After the measurements are obtained for each pool, the pooled level is used in an induced conditional logistic model:

2 $$ \frac{e^{\beta {v}_k^1}}{e^{\beta {v}_k^1}+{e}^{\beta {v}_k^0}} $$

where ${v}_k^1$ denotes the pooled (aggregate or sum of) exposure level (the measured level times *g*) from the *k* ^*th*^ case pool and ${v}_k^0$ denotes the pooled exposure level from the corresponding controls. The above model can be extended to incorporate confounders and effect modifiers (in limited capacity). It can be used to obtain maximum likelihood estimate of relevant parameters and associated SE. Consequently, standard statistical software can be employed to analyze data from a matched case control design with pooled exposure.

For a general model with multiple covariates *U*_*1*_ *, U*_*2*_ *, …, U*_*q*_ and an outcome of interest *D*, the contribution of the *i* ^*th*^ matched pair (1:1 matched design) is given by the following conditional logistic model:

3 $$ \frac{e^{\beta_1{u}_{1i}^1+{\beta}_2{u}_{2i}^1+\dots +{\beta}_q{u}_{qi}^1}}{e^{\beta_1{u}_{1i}^1+{\beta}_2{u}_{2i}^1+\dots +{\beta}_q{u}_{qi}^1}+{e}^{\beta_1{u}_{1i}^0+{\beta}_2{u}_{2i}^0+\dots +{\beta}_q{u}_{qi}^0}} $$

where ${u}_{ji}^1$ denotes the observed value of the covariate *U*_*j*_ for case and ${u}_{ji}^0$ denotes the observed value of the covariate *U*_*j*_ for control, *j = 1, 2, …, q*. Here, *β*_*j*_ denotes the log odds ratio (OR) associated with unit increase in the covariate *U*_*j*_. Denoting the pooled covariates as *V*_*1*_ *, V*_*2*_ *, …, V*_*q*_ and *k* indicating the pool, the pooled model is given by:

$$ \frac{e^{\beta_1{v}_{1i}^1+{\beta}_2{v}_{2i}^1+\dots +{\beta}_q{v}_{qi}^1}}{e^{\beta_1{v}_{1i}^1+{\beta}_2{v}_{2i}^1+\dots +{\beta}_q{v}_{qi}^1}+{e}^{\beta_1{v}_{1i}^0+{\beta}_2{v}_{2i}^0+\dots +{\beta}_q{v}_{qi}^0}} $$

where ${v}_{jk}^1$ denotes the pooled (aggregate or sum of) exposure level (the measured level times *g*) from the *k* ^*th*^ case pool and ${v}_{jk}^0$denotes the pooled exposure level from the corresponding controls, *j = 1, 2, q*. The details can be found in [30]. This approach is further clarified with a concrete example in the next sections.

**Fig. 2:**
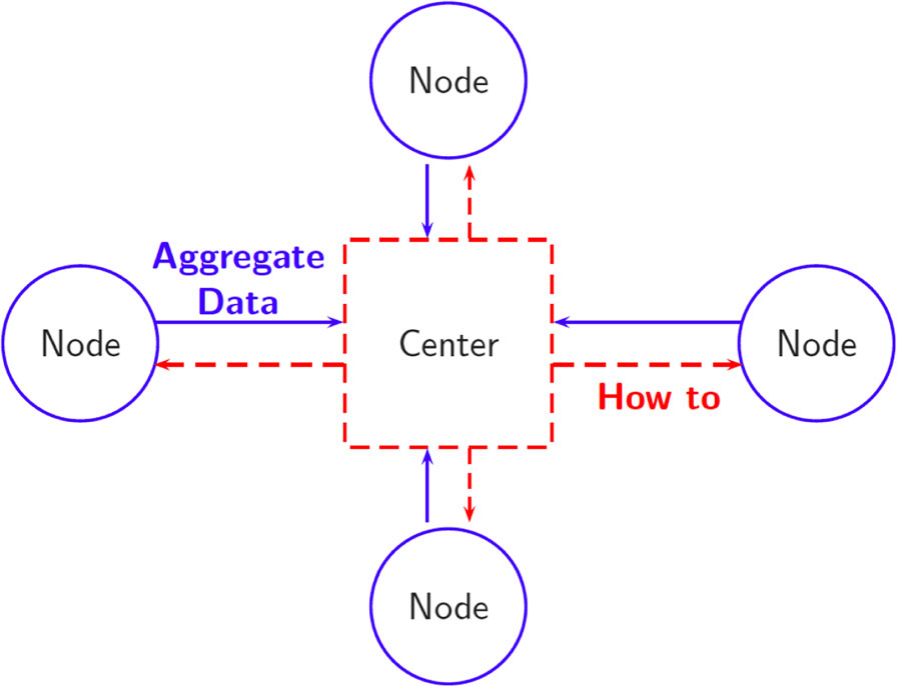
Schematic of distributed data. The analytical center is indicated by dashed red square and the nodes are indicated by solid blue circles. The arrows indicate the flow of information: the dashed red arrows represent the flow of instructions from center to nodes and the solid blue arrows represent the flow of aggregate data from the nodes to the center. Since the aggregate data do not reveal individual-level covariate combinations, and the center does not own microdata, the information flow is preserves data confidentiality

**Fig. 3:**
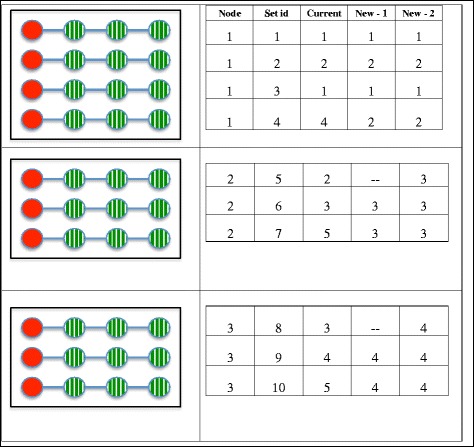
Schematic of pooling strategy for a 1:3 matched case-control study with three nodes, numbered 1–3 and 10 matched sets distributed over these nodes. For each matched set, case is indicated by filled red circle, three matched controls are indicated by shaded green circles. A line joins the case and controls in each matched stratum. Boxes represent the nodes. In the table on the right hand side, the first column indicates the node number and the second column indicates the matched set number. The 3rd column titled “Current” indicates the pool id if the current strategy of Saha-Chaudhuri and Weinberg (2017) is applied directly. The 4th and 5th column indicates the pool ids for two variants of the proposed within-node pooling strategy. In “New - 1” (4th column), poolsize g = 2 is used. The first two pools are formed within node 1, third pool is formed within node 2 and the fourth pool is formed within node 3. Since g = 2 is used and nodes 2 and 3 have odd number of matched sets, within-node pooling leads to exclusion of one matched set each from node 2 and node 3. In Scheme 2 (4th column, titled “New - 2”), poolsizes g = 2 and 3 are used. The first two pools are formed within node 1 (g = 2), third pool is formed within node 2 (g = 3) and the fourth pool is formed within node 3 (g = 3). Consequently, all participant data were used for analysis. Secure summation is not required for within-node stratified pooling as only the aggregate covariate levels are released from the nodes

To formalize the application of pooling in distributed data setting, we consider a horizontally partitioned data network with multiple nodes and one analytical center. In such a setting, each node holds covariate information of only a subset of participants (Fig. 1) and confidentiality issues prohibit data sharing and creation of a single analytical file encompassing individual-level data from all nodes. Typically, nodes are allowed to share masked data including summary tables, but not individual covariate combination of participants even with the AC (Fig. 2). In the toy example in Fig. 3, we show a horizontally partitioned structure with three nodes and 10 matching strata. Suppose a conditional logistic regression model similar to eq. (1) is postulated to assess association between a covariate *U* and a binary outcome *D*. The relevant application of pooling in this setting is in data aggregation or “virtual pooling” rather than pooling of biospecimens. Aggregation masks individual covariate combinations and hence could be utilized in this setting for estimation of association OR of relevant exposure(s) while protecting privacy of the participants. A direct application of pooling would then require formation of pools of size *g* across nodes. Following this, nodes will be required to work together and aggregate covariates for subjects in the pools and pass the pooled covariate levels to the AC. The AC could then use the aggregated levels in a conditional logistic regression model (similar to eq. (2)) to estimate the relevant parameters.

Such a scheme is logistically problematic for distributed data. Application of pooling to aggregate covariates [30] in this setting would require formation of pools across nodes. Consequently, data aggregation would not be possible without additional confidentiality measures such as secure computation [14]. Even with additional security measures, this approach is at best, cumbersome, and at worst nightmarish, for creation of all necessary aggregates in a distributed data setting (column termed “Current” in Fig. 3).

Rather than such unconditional grouping of participants’ data distributed over multiple nodes, we propose data pooling with stratification on node. In our proposed approach, instead of creating pools overall, each node creates their own pools, by randomly partitioning their matched sets into groups of size *g* and then aggregating the covariates within each set. These aggregate (summed) values are then sent to the AC for statistical analysis of the induced conditional logistic model similar to eq. (1). Due to application of within-node pooling, only aggregate covariate values are shared outside the nodes. The analysis can be done as before using a conditional logistic regression modeling approach. However, as shown in Fig. 3 (column “New - 1”), a limitation of this approach is that when nodes do not have a common divisor, use of a single *g* could lead to exclusion of multiple matched sets. This can be easily remedied by using two distinct values for *g* as shown in Fig. 3 where *g =* 2 and 3 were used (column “New - 2”).

The choice of the poolsize *g* is dictated primarily by the privacy rules. For example, in CNODES, many provinces require that the value of the descriptive statistics that is based on fewer than 5 participants will not be revealed. In such a situation, *g > 5* is appropriate. On the other hand, a large value of *g* would lead to reduction of statistical power [30, 31]. Therefore, the choice of *g* should balance the two issues of privacy restriction and realization of adequate statistical power. There are no other restrictions.

Confounders can be accommodated by summing the values within each of the pools (Table 1). However, in an epidemiologic setting, inclusion of EMs is potentially problematic [30–32]. In contrast, EMs can be handled easily in a distributed data setting because individual covariates are available at the nodes. Beyond within-node outcome stratification, we do not require additional stratification for EMs. Simply conditioning on the outcome status within nodes will allow us to treat the terms involving an EM as confounder terms and enable us to estimate ORs that involve EMs (both the main effect and the interaction). Similar to a stratum-specific confounder, one cannot estimate the main effect of a stratum-specific EM, but the effects of a subject-specific EM can be estimated using the proposed approach for distributed data. Additionally, transformation of variables can be handled easily by appropriately aggregating the transformed variables. Derived variables, such as body mass index and creatinine-corrected urinary levels, can also be accommodated by calculating the index and then summing across individuals. Similar to [30], our approach for data privacy can also accommodate a *1:M* *(M > 1)* matched and *N:M (N > 1, M > 1)* design.

**Table 1:** Pooled data passed from a node to the Analytical Center. We assume a 1:1 matched design. Here V^**1**^_i(1)_ (V^**2**^_i(1)_)denotes the pooled level of variable 1 (variable 2) for the i^th^ case pool and V^1^_i(0)_ (V^2^_i(0)_)denotes the pooled level of variable 1 (variable 2) for the i^th^ control pool within the node, *i* = 1, 2, …, k, where each pool consists of either *g* cases or *g* matched controls

Pool id	Pooled Covariate V_1_		Pooled Covariate V_2_		Pooled Covariate …	
	Case	Control	Case	Control	Case	Control
1	V^1^_1(1)_	V^1^_1(0)_	V^2^_1(1)_	V^2^_1(0)_	⋮	⋮
⋮	⋮	⋮	⋱	⋮	⋮	⋮
i	V^1^_i(1)_	V^1^_i(0)_	V^2^_i(1)_	V^2^_i(0)_	⋮	⋮
⋮	⋮	⋮	⋱	⋮	⋮	⋮
k	V^1^_k(1)_	V^1^_k(0)_	V^2^_k(1)_	V^2^_k(0)_	⋮	⋮

Thus, in a distributed data setting with a matched design, it is possible to assess association of the exposure with the outcome using pooled covariate determinations. The pooled model is simply a conditional logistic regression model with pooled or aggregate covariate levels instead of individual level covariates. Hence, in addition to the parameter estimate, we can estimate the SE, confidence interval and associated *p*-value for the model covariates (including transformations), except for stratum-specific variables. Consequently, model selection involving confounders, different transformations of the variables, and EMs will be possible. Two nested and competing models, fit using pooled data, can be compared using a likelihood ratio test or AIC.

## Results

### Simulation example^2^

Our simulation is motivated by a recent study of incretin-based drugs and congestive heart failure (CHF), which used a matched case-control design [33] nested within a cohort. In the study, the authors used data from United Kingdom’s Clinical Practice Research Datalink [34] and matched 1118 cases to ≤ 20 controls per case for a total of 17,626 controls. Matching factors were age, duration of treated diabetes, calendar year, and time since cohort entry. For simplicity, we considered a matched case-control design with 1020 matched sets, each matching 10 controls per case. To mimic a horizontally portioned data, we assumed that there are 5 nodes with 120, 180, 180, 240 and 300 matched sets, respectively. We simulated 500 datasets for each parameter combination and compared the proposed within-node pooling with the standard conditional logistic regression without pooling.

The primary exposure U was log normally distributed and was assumed to be a confidential variable. The secondary exposure X was binary with a prevalence of 0.4 and independent of U. A confounder Z_1_ was distributed as normal with a correlation of 0.35 with log(U). A continuous EM Z_2_ was generated as standard normal independent of U, X and Z_1_. Stratum-specific intercepts α_i_ for the i^th^ matched sets were generated as normal with mean of −3.0 and SD of 2. Node-specific intercepts ν_k_ for the k^th^ node were generated from a standard normal distribution with the assumption that the disease prevalence decreased with the number of matched strata resulting in smallest node with the highest baseline disease prevalence and largest node with the lowest baseline prevalence.

Finally, for the, the following model was considered for generating a binary disease outcome:

4 $$ \mathrm{logit}\Pr \left({D}_{kij}=1|\ U,X,{Z}_1,{Z}_2\right)={\alpha}_i+{\upsilon}_k+\beta\ U+\gamma\ X+\delta\ {Z}_1+\omega\ {Z}_2+\vartheta\ U\ {Z}_2 $$

where *D*_*kij*_ denotes the outcome of the j^th^ participant of the i^th^ matched set from the k^th^ node.

It can be shown that the conditional logistic regression model in this setting takes the form:

5 $$ \frac{e^{\beta {u}_1^1+\gamma {x}_1^1+\delta {z}_{11}^1+\omega {z}_{12}^1+\vartheta {u}_1^1{z}_{12}^1}}{e^{\beta {u}_1^1+\gamma {x}_1^1+\delta {z}_{11}^1+\omega {z}_{12}^1+\vartheta {u}_1^1{z}_{12}^1}+\sum_{J=2}^{M+1}{e}^{\beta {u}_j^0+\gamma {x}_j^0+\delta {z}_{j1}^0+\omega {z}_{j2}^0+\vartheta {u}_j^0{z}_{j2}^0}} $$

where ${u}_1^1,{x}_1^1,{z}_{11}^1,{z}_{12}^1\kern0.5em$denotes the covariate for the case and ${u}_j^0,{x}_j^0,{z}_{j1}^0,{z}_{j2}^0\kern0.5em$denotes the covariates for the j^th^ matched control, j = 2, …, M + 1 (= 11).

To demonstrate feasibility of our approach, we compared within-node pooled analysis with unpooled analysis. For pooled analysis, we considered within-node pools of sizes: g = 4, 6, 10 conditional on the outcome status, leading to 255, 170 and 102 matched strata. In contrast to the existing methods for pooled analysis, for distributed data, continuous effect modifiers can be included in the model as follows: we treat each additional term involving the EM as confounders and aggregate the appropriate functions (e.g., aggregate *Z*_2_ and *U Z*_2_ for the subjects in the same pool). The resulting model for the pooled covariates is of the form:

6 $$ \frac{e^{\beta \left({\sum}_{k=1}^g{u}_{1(k)}^1\right)+\gamma \left({\sum}_{k=1}^g{x}_{1(k)}^1\right)+\delta \left({\sum}_{k=1}^g{z}_{11(k)}^1\right)+\omega \left({\sum}_{k=1}^g{z}_{12(k)}^1\right)+\vartheta \left({\sum}_{k=1}^g{u}_{1(k)}^1{z}_{12(k)}^1\right)}}{e^{\beta \left({\sum}_{k=1}^g{u}_{1(k)}^1\right)+\gamma \left({\sum}_{k=1}^g{x}_{1(k)}^1\right)+\delta \left({\sum}_{k=1}^g{z}_{11(k)}^1\right)+\omega \left({\sum}_{k=1}^g{z}_{12(k)}^1\right)+\vartheta \left({\sum}_{k=1}^g{u}_{1(k)}^1{z}_{12(k)}^1\right)}+{\sum}_{j=2}^{M+1}{e}^{\beta \left({\sum}_{k=1}^g{u}_{i(k)}^0\right)+\gamma \left({\sum}_{k=1}^g{x}_{i(k)}^0\right)+\delta \left({\sum}_{k=1}^g{z}_{i1(k)}^0\right)+\omega \left({\sum}_{k=1}^g{z}_{i2(k)}^0\right)+\vartheta \left({\sum}_{k=1}^g{u}_{i(k)}^0{z}_{i2(k)}^0\right)}} $$

where ${u}_{1(k)}^1,{x}_{1(k)}^1,{z}_{11(k)}^1,{z}_{12(k)}^1$denotes the covariates for the k^th^ case in the pool (poolsize = g) and ${u}_{j(k)}^0,{x}_{j(k)}^0,{z}_{j1(k)}^0,{z}_{j2(k)}^0$ denotes the covariates for the j^th^ matched control, j = 2, 3, …, M + 1. Note that, in our proposed approach, participants providing data in a pool belong to the same node.

We considered several combinations of parameter values and show the results for the following set: *β* = 0.3 (corresponds to OR = 1.35), *γ* = 0.2 (OR = 1.22), *δ* = 0.15 (OR = 1.16), *ω* = 0.09 (OR = 1.09) and *ϑ* = 0.05 (OR = 1.05). We compared the average parameter estimate, average model-based SE (ModelSE), Monte Carlo SE (EmpSE) and coverage probability out of 500 simulations for unpooled (standard conditional logistic regression) and pooled conditional logistic regression with *g* = 4, 6, 10 for each parameters in the model (5 and 6). We define EmpSE as the standard deviation of the parameter estimates over the 500 simulations and ModelSE is the average of the 500 estimated SEs from the conditional logistic regression model. The results are summarized in Table 2.

**Table 2:** Parameter estimates between standard analysis and pooled analysis with different pool sizes for a binary outcome with a matched design. See Results section for detailed simulation setting. In addition, model-based SE (ModelSE), the Monte Carlo Standard Error (EmpSE) and coverage (nominal: 0:95) are also shown

Parameters	Unpooled	Pooled		
		*g* = 4	*g* = 6	*g* = 10
*β* = 0.3				
Estimate	0.301	0.303	0.307	0.336
EmpSE	0.014	0.022	0.028	0.077
ModelSE	0.014	0.022	0.029	0.060
Coverage	0.958	0.956	0.964	0.964
*γ* = 0.2				
Estimate	0.202	0.204	0.207	0.234
EmpSE	0.077	0.101	0.128	0.219
ModelSE	0.076	0.100	0.122	0.199
Coverage	0.954	0.956	0.944	0.952
*δ*= 0.15				
Estimate	0.149	0.150	0.150	0.165
EmpSE	0.037	0.049	0.062	0.104
ModelSE	0.037	0.049	0.060	0.098
Coverage	0.952	0.958	0.952	0.960
*ω* = 0.09				
Estimate	0.088	0.088	0.091	0.104
EmpSE	0.050	0.067	0.080	0.134
ModelSE	0.049	0.063	0.076	0.123
Coverage	0.964	0.936	0.942	0.954
*ϑ* = 0.05				
Estimate	0.050	0.051	0.051	0.054
EmpSE	0.013	0.018	0.023	0.039
ModelSE	0.013	0.018	0.022	0.037
Coverage	0.954	0.944	0.952	0.944

We see that in general, pooled results are similar to unpooled results, with a tendency to show slight bias away from the null, especially with increasing pool size, as expected given the reduction in the number of pooling sets analyzed. The model-based SE for pooled analysis was also slightly inflated as compared to model-based SE for unpooled analysis. However, the coverage for the 95% confidence interval for pool sizes 4 and 6 was in line with the nominal level, although slightly higher for poolsize 10.

### Real data example

We demonstrate our approach by creating a matched design to study the association of obesity (outcome of interest) with diastolic blood pressure based on the 2009–2010 cycle of the National Health and Nutrition Examination Study. The data (*N* = 5858 subjects with complete records, with 877 obese individuals) has been used for demonstration purposes [26] and is freely available.^3^ In addition to variables collected for the study, the dataset also includes a pseudo stratum variable that was used as units for sampling to demonstrate survey-weighted logistic regression. The covariates used for our analysis were gender, age at screening in years, stratum, Diastolic Blood Pressure, can walk or bike to work (yes/no), vigorous recreational activity (yes/no), moderate recreational activity (yes/no) and moderate work activity (yes/no). To mimic a distributed data, we considered that strata are nodes (15 strata) and each stratum cannot share the data of individual participant. We created a matched case-control study by matching 877 cases with 877 controls with respect to age (≤ 35 years, 31–60 years, 60 years and older), gender and stratum. For pooled analysis, we created within-stratum pools of size g = 4 and one stratum required the exclusion of one matched pair, which we selected at random. The results are displayed in Table 3.

**Table 3:** Comparison between pooled analysis and unpooled analysis of matched case-control study on obesity. The column marked “Standard CLogit” displays results of a standard conditional logistic regression analysis for the matched case-control study design. The column marked “Pooled CLogit” displays results of pooled conditional logistic regression analysis with within-stratum pooling and poolsize g = 4. The OR and 95% confidence interval (CI) for the covariates are includes

Variable	OR (95% CI)	
Analysis	Standard CLogit	Pooled CLogit
DBP	1.007 (0.999, 1.016)	1.005 (0.996, 1.014)
Can walk or bike to work	1.349 (1.068, 1.702)	1.357 (1.055, 1.745)
Vigorous Recreational Activity	1.710 (1.263, 2.315)	1.955 (1.352, 2.827)
Moderate Recreational Activity	1.289 (1.043, 1.592)	1.270 (1.016, 1.586)
Moderate work activity	0.853 (0.693, 1.049)	0.813 (0.653, 1.013)

The OR estimates based on the pooled conditional logistic regression model are in line with the unpooled estimate. The 95% CIs from the pooled model were similar to those computed using the model based on individual-level covariates. While it is perplexing to see that both moderate and vigorous recreational activities are associated with obesity, the same conclusion holds whether the pooled approach or the unpooled approach is used. Moreover, an unmatched analysis accounting for simulated clusters (stratum) and simulated survey-weights also showed similar results [26].

## Discussion

In this manuscript, we introduced an innovative application of specimen pooling for analyzing confidential distributed data. Large epidemiologic studies often combine data from various sources to study associations between covariates and an outcome of interest. For example, to examine post-marketing safety signal of marketed drugs, multiple registries may be combined. Large datasets are needed to assess associations of small magnitude as are typical in observational settings. Full covariate sharing between the nodes and AC would be ideal for analytical flexibility, but is not possible when there are concerns over data disclosure and identification of the participants. Existing policies and regulations often prohibit data sharing beyond the owners of the nodes or registries. Alternate methods include an aggregate table approach, distributed regression, score-based methods and meta-analysis [17]. However, many of these approaches share limitations such as, modeling inflexibility, collapsing of variables resulting in loss of information, large potential statistical burden on nodes and analytical complexity. In particular, meta-analysis, the most widely used of these approaches, requires that each node conduct their own analysis, imposing significant statistical burden on the nodes. Moreover, analytical exploration is severely limited for meta-analysis as post-hoc analyses are difficult to carry out without requiring participating sites to repeat analyses. Subgroup analysis is also operationally difficult for meta-analysis.

In contrast, our proposed approach of within-node pooling for matched case-control design retains many advantages of analyses with individual-level data. The participating nodes are only required to aggregate the covariates according to a given poolsize and using outcome-stratification. Within-node pooling is also advantageous when nodes are considered as potential EM. Once the aggregation is performed, the nodes can send the aggregate covariates (summed covariate vectors) to the AC without compromising data confidentiality and without requiring any further statistical analyses on their part. Even when a large set of models is considered for analysis, the nodes are only required to do aggregation and nothing more. Data analyses, including model selection (as possible based on the pre-determined models and aggregated covariates), pre-defined subgroup analysis could be done without any statistical burden on the nodes. As such, our approach is closest to analysis with full covariate sharing as compared to the other methods, and with added protection for data disclosure. In this manuscript, we focused only on a matched case-control design; however, the approach is also applicable for an unmatched design [25, 29].

As shown in the simulation study, the proposed pooling technique allows consistent estimation of effects associated with a primary exposure and confounders. In addition, by recognizing that the terms involving the EM can be treated as additional confounders, effects of EM can be assessed. Furthermore, transformation of variables, such as log or polynomials, can be accommodated and in each case, the relevant parameter(s) can be tested using a Wald t-test or likelihood ratio test, allowing researchers to perform model selection. Since the induced pool-based model is a conditional logistic regression model, analysis does not require any novel tool. Standard statistical software can be used for analysis. Beyond aggregation of covariates, our approach does not impose any computational burden on nodes with limited statistical capability. This is in stark contrast to the existing approaches [35, 36]. When, in addition to confidential data, banked biospecimens are available for association study, specimen pooling and data pooling can both be incorporated in the study to protect confidentiality and make effective use of valuable biospecimen and monetary resources. However, researchers should be careful about the design and the implications of specimen pooling, in particular for estimating effect modification and/or transformation of variables.

The approach does have limitations. While pooling protects data privacy, there may be certain situations where confidentiality may not be fully guaranteed using our approach, especially when smaller pool size is used. As an extreme example, if the microdata consists of all binary covariates, and a pool size of 2 is used, it is easy to deduce the individual covariate values, when all the pooled covariate values are either 0 or 2. While a confidential microdata with all binary covariate will be relatively rare, other techniques can be adapted for analysis of such a dataset [36]. When several pre-specified models are postulated for the data, our approach can be used efficiently by requiring that nodes send all relevant aggregate covariate values. However, if a model is decided a posteriori, and the new model includes any new covariates (including transformations and interactions), then nodes need to get involved again to send the appropriate aggregate values back to the AC. However, this is still relatively straightforward as compared to using a new model for meta-analysis where node would be required to redo the entire analysis. Another limitation is that our proposed approach can be used only for matched or unmatched case-control design with a logit link. Currently no aggregation-based method exists for survival models. For the choice of poolsize, it is important to balance logistical complexity and maximal data use. Based on extensive simulations, we recommend the use of poolsizes between 3 and 6 and possibly use of two different poolsizes for a study.

## Conclusion

In summary, we have shown here that a specimen pooling approach can be adapted successfully for virtual data pooling as a privacy-preserving analytical tool for analysis of confidential data. Our proposed pooling approach uses only aggregated covariate information, thereby making it impossible to link individual data to individual participants. This general strategy can be used in conjunction with data from either a matched or an unmatched case-control study design [25, 29, 30]. In this context, pooling is equivalent to microaggregation, which has been used extensively as a statistical disclosure limitation technique for linear models for confidential financial and/or survey data, among others. [37–39] However, microaggregation has not previously been proposed for logistic regression. We demonstrated the feasibility of this technique for analysis of confidential data using simulations and a real data example. Other pooling techniques could also be adapted for analysis of confidential data, in fact one of the primary use of specimen pooling is in infectious disease setting where it has helped to protect patient privacy. This approach could expand the capabilities of epidemiologic research involving distributed, confidential datasets.

## Abbreviations

AC: analytical center
AIC: Akaike Information Criteria
CHF: congestive heart failure
CNODES: Canadian Network for Observational Drug Effect Studes
CPRD: Clinical Practice Research Datalink, UK
EM: effect modifier
FDA: Food and Drug Administration, USA
OR: odds ratio
SE: standard error

## Footnotes

1 We have used the clogit function in the programming language R to fit a conditional logistic regression model to data from a matched case control design.

2 All simulations and statistical analyses were performed using the programming language R. The built-in R function “clogit” was used to estimate parameters of eq. (4) and eq. (5). R codes are available upon request from the corresponding author.

3 Available from: https://sites.google.com/site/paramitasaharesearch/data/
